# A novel visual illusion paradigm provides evidence for a general factor of illusion sensitivity and personality correlates

**DOI:** 10.1038/s41598-023-33148-5

**Published:** 2023-04-22

**Authors:** Dominique Makowski, An Shu Te, Stephanie Kirk, Ngoi Zi Liang, S. H. Annabel Chen

**Affiliations:** 1 School of Social Sciences, Nanyang Technological University, Singapore, Singapore; 2 LKC Medicine, Nanyang Technological University, Singapore, Singapore; 3 National Institute of Education, Singapore, Singapore; 4 Centre for Research and Development in Learning, Nanyang Technological University, Singapore, Singapore; 5 Present Address: School of Psychology, University of Sussex, Brighton, UK

**Keywords:** Psychology, Human behaviour, Cognitive neuroscience, Consciousness, Perception

## Abstract

Visual illusions are a gateway to understand how we construct our experience of reality. Unfortunately, important questions remain open, such as the hypothesis of a common factor underlying the sensitivity to different types of illusions, as well as of personality correlates of illusion sensitivity. In this study, we used a novel parametric framework for visual illusions to generate 10 different classic illusions (Delboeuf, Ebbinghaus, Rod and Frame, Vertical-Horizontal, Zöllner, White, Müller-Lyer, Ponzo, Poggendorff, Contrast) varying in strength, embedded in a perceptual discrimination task. We tested the objective effect of the illusions on errors and response times, and extracted participant-level performance scores (n=250) for each illusion. Our results provide evidence in favour of a general factor underlying the sensitivity to different illusions (labelled Factor *i*). Moreover, we report a positive link between illusion sensitivity and personality traits such as Agreeableness, Honesty-Humility, and negative relationships with Psychoticism, Antagonism, Disinhibition, and Negative Affect.

## Introduction

Visual illusions are fascinating stimuli capturing a key feature of our neurocognitive systems. They eloquently show that our brains did not evolve to be perfect perceptual devices providing veridical accounts of physical reality, but integrate prior knowledge and contextual information - blended together in our subjective conscious experience^1^. Despite the long-standing interest within the fields of visual perception^2–4^, consciousness science^5,6^, and psychiatry^7–10^, several important issues remain open.

One area of contention concerns the presence of a common mechanism underlying the effect of different illusions^11,12^. While early research has suggested a common factor of illusion sensitivity indexed by overall vision proficiency^13,14^, recent empirical studies observed at most weak correlations between inter-individual resistance to distinct illusions^15,16^. The existence of dispositional correlates of illusion sensitivity has also been controversial, with evidence suggesting a lower illusion sensitivity in patients with schizophrenia and autism^7–9,16,17^, as well as individuals with stronger aggression and narcissism traits^18,19^.

Although the nature of the processes underlying illusion perception - whether related to low-level features of the visual processing system^8,20^ or to top-down influences^5,21^ - remains debated, a growing body of literature proposes to conceptualize illusions under the Bayesian brain hypothesis^22^. In this context, illusions are conceptualized as non-veridical perceptual experiences that result from giving ample weight to prior knowledge to minimize prediction error in the face of biasing sensory evidence. The predictive coding account further provides an explanation regarding the observations from clinical populations. Certain dispositional traits or characteristics (e.g., psychoticism) are seen as driven by alterations in the system’s metacognitive components^23^, resulting in an underweighting of priors during perceptual inferences, and manifesting as a decreased sensitivity to illusions^24^.

Despite strong theoretical foundations and hypotheses, the empirical evidence remains scarce, clouded by methodological hurdles. For instance, one key challenge can be found in the difficulty of adapting visual illusions to an experimental setting, which typically requires the controlled modulation of the specific variables of interest. Instead, existing studies typically use only one or a small subset of illusion types, with few contrasting conditions, restricting the findings’ generalizability^12,20,25^. Moreover, conventional paradigms often focus on the participants’ subjective experience, by asking them the extent to which they perceive two identical targets as different^26^, having them estimate the targets’ physical properties^27^, or through the method of adjustment, which involves having them adjust the targets to perceptually match a reference stimulus^16,28–30^. This reliance on meta-cognitive judgements about one’s subjective experience likely distorts the measurand, limiting the ability to reliably obtain more direct and objective measures of illusion sensitivity^31^. While some recent efforts have some made to implement more empirically rigorous paradigms^32^, most of the applied manipulations only focus on varying the physical dimensions of the illusion’s target features without modulating its contextual elements, hence limiting the variability in the illusory effects of the stimuli presented. Furthermore, such prior studies have typically generated stimuli whose targets’ physical attributes vary over a relatively narrow range, thus further constraining the reliability of their findings. As such, it is possible that the recent evidence reported against a common factor of illusions could be due to the low stimulus variance instead of a true reflection of a lack of common mechanism.

**Figure 1:**
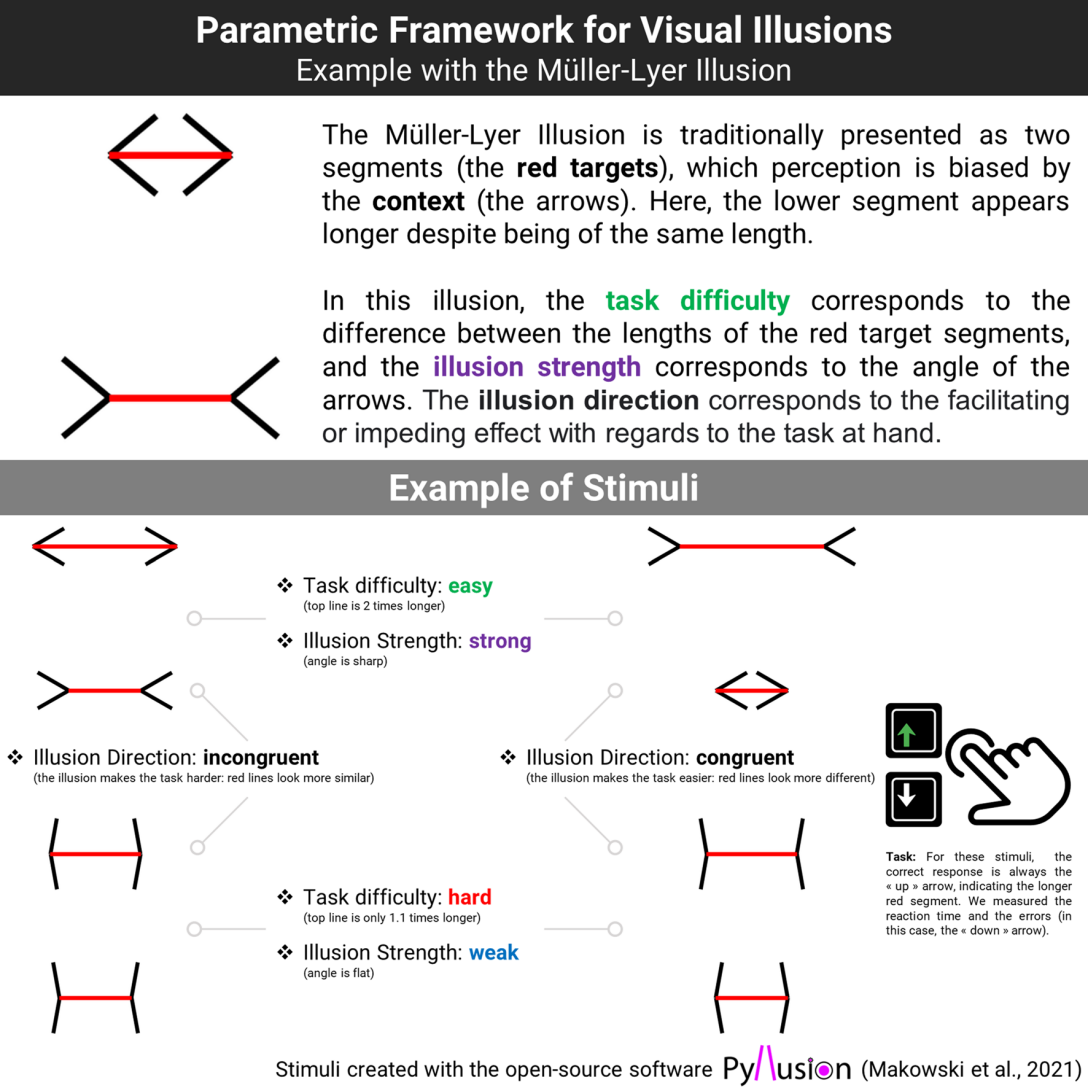
The parametric framework for visual illusions (Makowski et al., 2021) applied to the Müller-Lyer illusion (above). Below are examples of stimuli showcasing the manipulation of two parameters, task difficulty and illusion strength.

To address these issues, we first developed a parametric framework to manipulate visual illusions that we implemented and made accessible in the open-source software *Pyllusion*^33^. This software allows us to generate different types of classic visual illusions with a continuous and independent modulation of two parameters: *illusion strength* and *task difficulty* (Fig. 1). Indeed, many visual illusions can be seen as being composed of *targets* (e.g., same-length lines), of which perception is biased by the *context* (e.g., in the Müller-Lyer illusion, the same-length line segments appear to have different lengths if they end with inwards vs. outwards pointing arrows). Past illusion studies traditionally employed paradigms focusing on participants’ subjective experience, by asking them the extent to which they perceive two identical targets as different^26^, or having them adjust the targets to match a reference stimulus relying only on their perception^16,28^. Alternatively, *Pyllusion* allows the creation of illusions in which the targets are objectively different (e.g., one segment is truly more or less longer than the other), and in which the illusion varies in strength (the biasing angle of the arrows is more or less acute).

This systematic calibration of the stimuli enables the creation of experimental tasks in which participants make perceptual judgments about the targets (e.g., which segment is the longest) under different conditions of objective difficulty and illusion strength. Moreover, the illusion effect can be specified as either “incongruent” (making the task more difficult by biasing the perception in the opposite way) or “congruent” (making the task easier). Although visual illusions are inherently tied to subjective perception, this framework allows a reversal of the traditional paradigm to potentially quantify the “objective” effect of illusions by measuring its behavioral effect (error rate and reaction times) on the performance in a perceptual task.

The aim of the present preregistered (https://osf.io/5d6xp) study is three-fold. First, we will test this novel paradigm by investigating if the effect of illusion strength and task difficulty can be manipulated continuously for 10 different classic illusions (Delboeuf, Ebbinghaus, Rod and Frame, Vertical-Horizontal, Zöllner, White, Müller-Lyer, Ponzo, Poggendorff, Simultaneous Brighness Contrast). Next, we will investigate the factor structure of illusion-specific performance scores and test the existence of a common latent factor of illusion sensitivity. Finally, we will explore how illusion sensitivity relates to demographic characteristics, contextual variables, and personality traits.

## Methods

### Ethics statement

This study was approved by the NTU Institutional Review Board (NTU IRB-2022-187) and all procedures performed were in accordance with the ethical standards of the institutional board and with the 1964 Helsinki Declaration. All participants provided their informed consent prior to participation and were incentivized after completing the study.

### Stimuli

**Figure 2:**
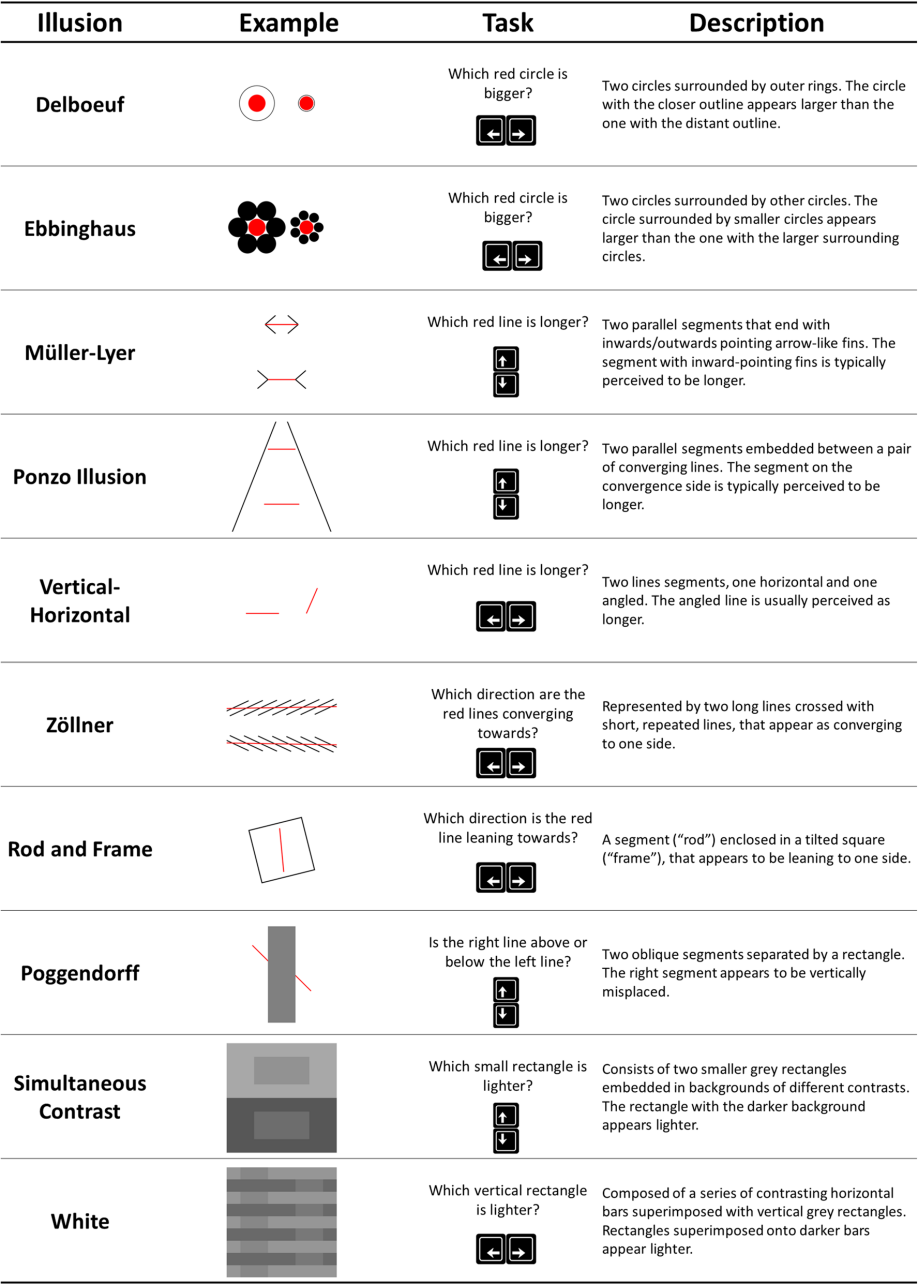
Ten different illusions were used as stimuli in a perceptual task, where participants had to answer as fast as possibly, without making errors, according to specific instructions. For each illusion type, two parameters were experimentally manipulated, (1) the task difficulty (e.g., how large was the difference between the bigger and the smaller red circles in the Delboeuf illusion), and (2) the illusion strength (e.g., the size of the black circles in the Ebbinghaus illusion).

We investigated the effect of 10 different classic illusions (Fig. 2). A pilot study (*n = 46*), of which a full description is available at https://github.com/RealityBending/IllusionGameValidation, was first conducted to determine a sensitive range of stimuli parameters. Then, for each of the 10 illusion types, we generated a total of 134 stimuli. These stimuli resulted from the combination of 15 equally-spaced levels of illusion *strength* (7 negative, i.e., congruent effects; 7 positive, i.e., incongruent effects; and 0) overlapped with 16 non-linearly spaced task *difficulty* levels (i.e., with an exponential, square or cubic spacing depending on the pilot results). For instance, a linear space of [0.1, 0.4, 0.7, 1.0] can be transformed to an exponential space of [0.1, 0.34, 0.64, 1.0], where 0.1 corresponds to the highest difficulty - i.e., the smallest objective difference between targets). For each illusion type, the stimuli were split into two series (56 and 72 stimuli per series) with alternating parameter values to maintain their homogeneity. Additionally, 6 stimuli per illusion type were generated for a practice series using parameters with more extreme variations (i.e., containing very easy trials to help cement the task instructions).

### Procedure

Participants were first given a brief demographic survey, which collected information regarding their age, gender, country of birth, ethnicity and highest attained education level. This was followed by a practice series of illusions, after which the first series of 10 illusion blocks was presented in a randomized order, with a further randomization of the stimuli order within each block. Following this first series of blocks, two personality questionnaires were administered, the *IPIP6*^34^ - measuring 6 “normal” personality traits (Extraversion, Openness, Conscientiousness, Agreeableness, Neuroticism and Honesty-Humility), and the *PID-5*^35^ - measuring 5 “pathological” personality traits (Disinhibition, Antagonism, Detachment, Negative Affect and Psychoticism). Next, the second series of 10 illusion blocks was presented (with new randomized orders of blocks and trials). In total, each participant underwent 1340 trials of which they had to respond “as fast as possible without making errors” (i.e., an explicit double constraint to mitigate the inter-individual variability in the speed-accuracy trade off) by pressing the correct arrow key (left/right, or up/down depending on the illusion type). For instance, in the Müller-Lyer block, participants had to answer which one of the upper or bottom target line was the longest. All trials were required to be completed within a single-session (total experiment duration: ~55 minutes). The task was implemented using *jsPsych*^36^ and was hosted on *Pavlovia* (https://pavlovia.org/). The set of instructions for each illusion type is available in the experiment code.

### Participants

Participants were recruited via *Prolific*, a crowd-sourcing platform recognized for providing high quality data^37^. The only inclusion criterion was a fluent proficiency in English to ensure that the task instructions would be well-understood. Participants were incentivised with a reward of about £7.50 for completing the task, which took approximately 50 minutes to finish. Demographic variables (age, gender, and ethnicity) were self-reported on a voluntary basis.

We excluded 6 participants upon inspection of the average error rate (when close to 50%, suggesting random answers), and reaction time distribution (when implausibly fast relative to the average RT distribution). For the remaining participants, we discarded blocks with more than 50% of errors (2.16% of trials), possibly indicating that instructions were misunderstood (e.g., participants focused on the shorter line instead of the longer one), and 0.76% trials with extreme response times (< 125 ms or > 4 SD above mean RT). Additionally, due to a technical issue, no personality data was recorded for the first eight participants.

The final sample included 250 participants (Mean age = 26.5, SD = 7.6, range: [18 - 69]; Sex: 48% females, 52% males).

### Data analysis

The first part of the analysis focused on modelling the effect of illusion strength and task difficulty on errors and response time (RT) separately for each illusion under a Bayesian framework. We started by fitting General Additive Models (GAMs), which can parsimoniously accommodate possible non-linear effects and interactions. Errors were analyzed using logistic mixed models (suited to estimate the error rate), and RTs of correct responses were analyzed using an ex-Gaussian family with the same fixed effects entered for the location $\mu$ (mean), scale $\sigma$ (spread) and tail-dominance $\tau$ of the RT distribution^38,39^.

**Figure 3:**
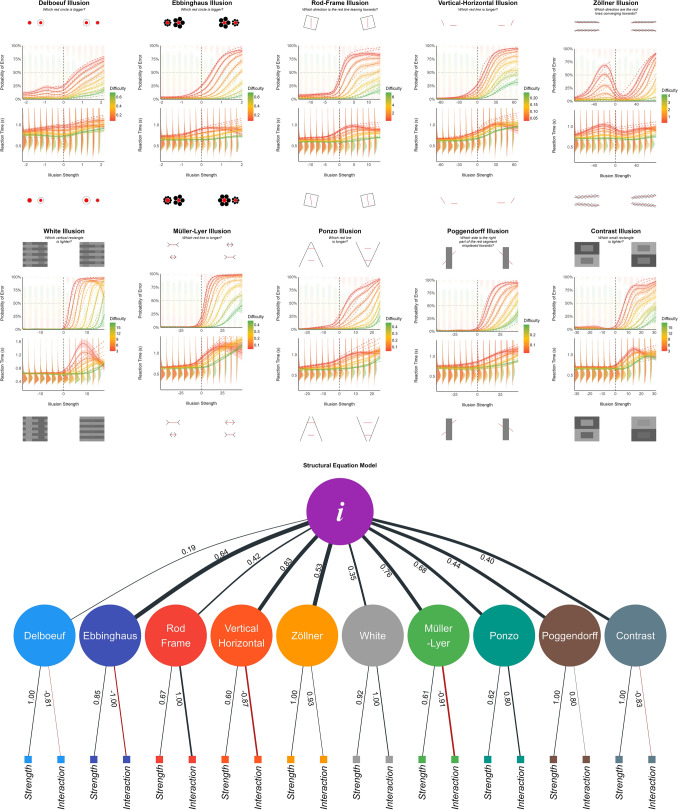
Top: the effect of illusion strength and task difficulty on the error rate and reaction time (RT) for each individual illusion. The solid line represents the General Additive Model (GAM), and the dashed line corresponds to its approximation via linear models. Descriptive data is shown with stacked dots (for which errors start from the top) and distributions for RTs. Negative values for illusion strength correspond to congruent (i.e., facilitating) illusion effects. Task difficulty (the objective difference between the targets of perceptual decision) levels are shown as colors, with lower values corresponding to harder trials. The results for each illusion are surrounded by 4 extreme examples of stimuli, corresponding to the hardest difficulty (on top) and the strongest illusion (on the right for incongruent illusions). Bottom: We extracted the effect slope of the illusion strength and its interaction with task difficulty for each participant. We fitted a Structural Equation Model (SEM) suggesting that these manifest variables group to first-level illusion-specific latent factors, which then load on a general factor of illusion sensitivity (Factor *i*).

Using GAMs as the “ground-truth” models, we attempted at approximating them using general linear mixed models, which can be used to estimate the effects’ participant-level variability (via random slopes). Following a comparison of models with a combination of transformations (raw, log, square root or cubic root; which are types of relationship commonly found in perceptual tasks) on the main predictors (task *difficulty* and illusion *strength*), we fitted the best model (based on their BIC and R2), and compared their output visually (Fig. 3). Note that the model comparison and the parameters used in the resulting models were not pre-registered.

The inter-individual variability in the effect of illusion strength and its interaction with task difficulty (diff) was extracted from the models and used as participant-level scores. We then explored the relationship of these indices across different illusions using exploratory factor analysis (EFA, to gain insights into the structure), and structural equation modelling (SEM, to model and test different hierarchical models), and tested the existence of a general factor of illusion sensitivity (Factor *i*).

Finally, for each of the individual illusion sensitivity scores (10 illusion-specific factors and the general Factor *i*), we tested the effect of contextual variables (screen size, screen refresh rate), demographic variables (sex, education, age), and personality traits. It should be noted that the measure of screen size used (measured using the number of pixels) is only a proxy of the true physical screen size.

The analysis was carried out using *R 4.2*^40^, *brms*^41^, the *tidyverse*^42^, and the *easystats* collection of packages^43–46^. As all the full results have been made available (see Data Availability), we will focus here on the significant results (based on the Bayes Factor *BF* or the Probability of Direction *pd*^47^).

### Significance statement

A novel paradigm to study the objective effect of visual illusions yielded evidence in favor of a common factor to visual illusions (Factor *i*) and a relationship between illusion resistance and maladaptive personality traits, such as antagonism, psychoticism and disinhibition.

## Results

### Effects of illusion strength and task difficulty

The best model specifications were $log(diff)*strength$ for Delboeuf; $sqrt(diff)*strength$ for Ebbinghaus; $log(diff)*log(strength)$ for Rod and Frame; $sqrt(diff)*sqrt(strength)$ for Vertical-Horizontal; $cbrt(diff)*strength$ for Zöllner; $diff*sqrt(strength)$ and $log(diff)*strength$ respectively for errors and RT in White; $sqrt(diff)*sqrt(strength)$ and $sqrt(diff)*strength$ respectively for errors and RT in Müller-Lyer; $cbrt(diff)*strength$ for Ponzo; $cbrt(diff)*sqrt(strength)$ and $cbrt(diff)*strength$ respectively for errors and RT in Poggendorff; and $sqrt(diff)*sqrt(strength)$ for Contrast. For all of these models, the effects of illusion strength, task difficulty and their interaction were significant.

For error rates, most of the models closely matched their GAMs counterpart, with the exception of Delboeuf (for which the GAM suggested a non-monotonic effect of illusion strength with a local minimum at 0) and Zöllner (for which theoretically congruent illusion effects were related to increased error rate). A specific discussion regarding these 2 illusions is available in the study documentation (part 1) at https://github.com/RealityBending/IllusionGameValidation.

For RTs, the GAMs suggested a consistent non-linear relationship between RT and illusion strength: as the illusion strength increases beyond a certain threshold, the participants responded faster. While this is not surprising (strong illusions are likely so effective in biasing perception that it is “easier”, i.e., faster, to make the wrong decision), the linear models were not designed to capture this - likely quadratic - pattern and hence are not good representatives of the underlying dynamics. As such, we decided not to use them for the individual scores analysis.

### Factor structure

Though imperfect, we believe that the random-slope models capture inter-individual differences with more accuracy (and also provide more conservative estimates due to shrinkage) than basic empirical scores, such as the total number of errors, or the average RT. Thus, for each illusion and within each participant, we extracted the effect of illusion strength and its interaction with task difficulty when the illusion effect was incongruent. These twenty participant-level scores were subjected to exploratory factor analysis (EFA). The Method Agreement Procedure^48^ suggested the presence of 7 latent factors. An oblique (*oblimin* rotation) factor solution explaining 66.69% of variance suggested separate dimensions for the effect of Zöllner, White, Poggendorff, Contrast, Ebbinghaus, Delboeuf, and a common factor for the parameters related to Müller-Lyer, Vertical-Horizontal, Ponzo and Rod and Frame. We submitted these factors to a second-level analysis and extracted two orthogonal (*varimax* rotation) factors. The first factor was loaded by all the previous dimensions with the exception of Delboeuf, which formed its own separate factor.

Finally, we tested this data-driven model (*m0*) against four other structural models using structural equation modelling (SEM): one in which the two parameters of each of the 10 illusions (illusion strength and interaction with task difficulty) loaded on separate factors, which then all loaded on a common factor (*m1*); one in which the parameters were grouped by illusion type (lines, circles, contrast and angle) before loading on a common factor (*m2*); one in which all the parameters related to strength, and all the parameters related to the interaction loaded onto two respective factors, which then loaded on a common factor (*m3*); and one in which there was no intermediate level: all 20 parameters loaded directly on a common factor (*m4*).

The model *m1*, in which the parameters loaded on a first level of 10 illusion-specific factors, which then all loaded on a common factor, significantly outperformed the other models. Its indices of fit ranged from acceptable to satisfactory (CFI = .92; SRMR = .08; NNFI = .91; PNFI = .74; RMSEA = .08), and all the specified effects were significant. The illusion-specific latent factors were loaded positively by the sensitivity to illusion strength, as well as by the interaction effect with task difficulty (with the exception of Delboeuf, Ebbinghaus, Vertical-Horizontal, Müller-Lyer and Contrast, for which the loading was negative). The general factor of illusion sensitivity, labelled Factor *i* (i- for illusion), explained 48.02% of the total variance of the initial dataset, and was strongly related to Vertical-Horizontal ($\beta _{std.}=0.83$), Müller-Lyer ($\beta _{std.}=0.76$), Ponzo ($\beta _{std.}=0.65$), Ebbinghaus ($\beta _{std.}=0.64$); moderately to Zöllner ($\beta _{std.}=0.53$), Poggendorff ($\beta _{std.}=0.44$), Rod and Frame ($\beta _{std.}=0.42$), Contrast ($\beta _{std.}=0.40$) and White ($\beta _{std.}=0.35$); and weakly to Delboeuf ($\beta _{std.}=0.19$). We then computed, for each participant, the score for the 10 illusion-specific factors and for the general Factor *i*.

It is important to note that these individual scores are the result of several layers of simplification: 1) the individual coefficient is that of simpler models that sometimes do not perfectly capture the underlying dynamics (especially in the case of Delboeuf and Zöllner); 2) we only used the models on error rate, which could be biased by the speed-accuracy decision criterion used by participants; 3) the structural equation model used to compute the scores also incorporated multiple levels of abstractions. Thus, in order to validate the individual scores, we computed the correlation between them and simple empirical scores, such as the average error rate and the mean RT in the task. This analysis revealed strong and significant correlations between each illusion-specific factor and the average amount of errors in its corresponding task. Moreover, each individual score was strongly associated with the average RT across multiple illusion types. This suggests that the individual scores obtained from the structural equation model do capture the sensitivity of each participant to visual illusions, manifesting in both the number of errors and long reaction times.

### Correlations with inter-individual characteristics

The Bayesian correlation analysis (with narrow priors centered around a null effect) between the illusion scores and contextual variables (screen size and refresh rate) provided weak evidence in favor of an absence of effect, with the exception of the two contrast-based illusions. Anecdotal ($BF_{10} = 2.05$) and moderate evidence ($BF_{10} = 4.11$) was found for a negative correlation between screen size and the sensitivity to the White and the Contrast illusion, respectively. To test whether this result could be an artifact related to the highly skewed screen size distribution (caused by very few participants with extreme screen sizes), we re-ran a robust correlation (with rank-transformed values), which provided even stronger evidence in favor of the effect existence ($BF_{10} = 28.19$, $BF_{10} = 4.31$ for White and Contrast, respectively).

**Figure 4:**
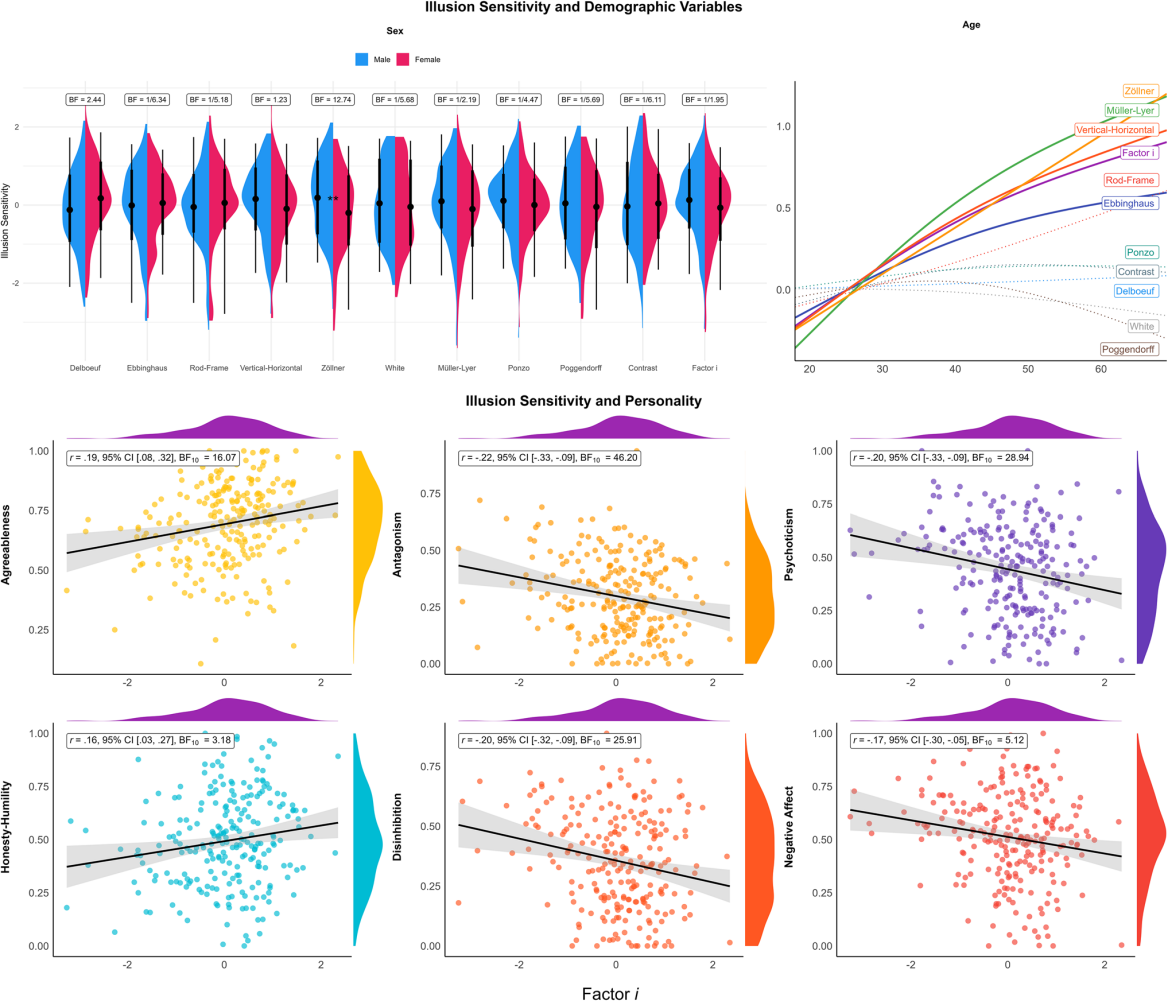
The upper plots show the illusion sensitivity scores as a function of sex and age (solid lines indicate significant relationships). Bottom plots show the correlation between the general factor (Factor *i*) of illusion sensitivity (on the x-axes) and personality traits.

The Bayesian t-tests on the effect of sex suggested anecdotal to moderate evidence in favour of the null effect for all scores, with the exception of the sensitivity to the Zöllner illusion, which was higher in males as compared to females ($\Delta =-0.37$, 95% CI [-0.62, -0.13], $BF_{10} = 12.74$). We fitted Bayesian linear models with the education level entered as a monotonic predictor (appropriate for ordinal variables^49^]) which yielded no significant effects. For age, we fitted two types of models for each score, one general additive models (GAM) and a 2nd order polynomial model. These consistently suggested a significant positive linear relationship between age and Factor *i* ($pd=100\%$), as well as the sensitivity to Müller-Lyer ($pd=100\%$), Vertical-Horizontal ($pd=100\%$), Zöllner ($pd=100\%$) and Ebbinghaus ($pd=99\%$) illusions (Fig. 4).

Regarding “normal” personality traits, Bayesian correlations suggested substantial evidence in favor of a positive relationship between *Honesty-Humility* and Zöllner ($BF_{10} > 100$), Vertical-Horizontal ($BF_{10} = 9.78$) and the Factor *i* ($BF_{10} = 4.00$); as well as between *Agreeableness* and Vertical-Horizontal ($BF_{10} = 25.06$), Ponzo ($BF_{10} = 4.88$) and the Factor *i* ($BF_{10} = 19.65$).

Regarding “pathological” personality traits, the results yielded strong evidence in favor of a negative relationship between illusion scores and multiple traits. *Antagonism* was associated with the sensitivity to Vertical-Horizontal ($BF_{10} > 100$), Müller-Lyer ($BF_{10} = 21.57$), Ponzo ($BF_{10} = 17.97$) illusions, and the Factor *i* ($BF_{10} = 55.45$); *Psychoticism* was associated with the sensitivity to Vertical-Horizontal ($BF_{10} = 66.63$) and Müller-Lyer ($BF_{10} = 35.59$) illusions, and the Factor *i* ($BF_{10} = 35.02$); *Disinhibition* was associated with the sensitivity to Vertical-Horizontal ($BF_{10} = 25.38$), Zöllner ($BF_{10} = 7.59$), Müller-Lyer ($BF_{10} = 5.89$) illusions, and the Factor *i* ($BF_{10} = 31.42$); and *Negative Affect* was associated with Zöllner ($BF_{10} = 62.04$), Vertical-Horizontal ($BF_{10} = 12.65$), Müller-Lyer ($BF_{10} = 3.17$), and the Factor *i* ($BF_{10} = 6.39$). The last remaining trait, *Detachment*, did not share any significant relationship with illusion sensitivity.

## Discussion

This study tested a novel illusion sensitivity task paradigm based on the parametric illusion generation framework^33^. Using the carefully generated stimuli in a perceptual decision task, we have shown that a gradual modulation of illusion strength is effectively possible across 10 different types of classic visual illusions. Increasing the illusion strength led to an increase in error likelihood, as well as the average and spread of RTs (but only up to a point, after which participants become faster at responding with the wrong answer). Using mixed models, we were able to statistically quantify the effect of illusions for each illusion and each participant separately. This important methodological step opens the door for new illusions-based paradigms and tasks to study the effect of illusions under different conditions and to measure illusion sensitivity using objective behavioral outcomes - such as accuracy or speed - instead of subjective meta-cognitive reports. This new and complementary approach will hopefully help address some of the longstanding literature gaps, as well as cement illusions as valuable stimuli for the study of cognition.

Our findings suggest that the sensitivity to 10 different types of visual illusions share a common part of variance, supporting the existence of a general factor of illusion sensitivity (Factor *i*). This result comes in a field of mixed findings. In fact, contrary to early studies on visual illusions, more recent research have generally not found any significant evidence for a common stable factor across illusions within individuals^12,15,16,20,50^. Instead, past findings suggest illusory effects are highly specific to the perceptual features of the illusions at stake^15,20^. It should be noted, however, that most of these studies were low-powered and/or relied on conventional paradigms, such as the adjustment procedure to measure the participants’ subjective perception. We believe that our study presents several methodological improvements, including statistical power (high number of trials per participant), homogeneous stimuli (with minimal and highly controlled features) and tasks (decision-making reaction-time task), and a more reliable participant-level score extraction method (based on random-factors models), which in our opinion contributed to the emergence of the common factor.

Finally, we found illusion sensitivity to be positively associated with “positive” personality traits, such as agreeableness and honesty-humility, and negatively associated with maladaptive traits such as antagonism, psychoticism, disinhibition, and negative affect. Although the existing evidence investigating links between illusion sensitivity and personality traits is scarce, these results are consistent with past findings relating pathological egocentric beliefs often associated with psychoticism^51^, to reduced context integration, manifesting in a tendency to separate objects from their surroundings when processing visual stimuli^19,51,52^. As such, the association between maladaptive traits and lower illusion sensitivity could be linked to a self-centered, decontextualized and disorganized information processing style. Conversely, the relationship between illusion sensitivity and adaptive personality traits is in line with the decreased field dependence (the tendency to rely on external cues in ambiguous contexts) associated with traits negatively correlated with agreeableness and honesty-humility, such as hostility, aggression and narcissism^18,19,53^.

Importantly, these findings highlight the relevance of illusions beyond the field of visual perception, pointing towards an association with high-level domain-general mechanisms. In particular, the evidence in favor of a relationship between maladaptive personality traits and illusion sensitivity is in line with clinical observations, in which a greater resistance to illusions have been reported among patients with schizophrenia^7,16,53^, especially in association with schizotypal traits such as cognitive disorganization^20,26^. While the search for the exact mechanism(s) underlying these links is an important goal of future research, our findings unlock the potential of illusion-based tasks as sensitive tools to capture specific inter-individual neuro-cognitive differences.

Future research is needed to address several limitations. One key question concerns the relationship of illusion sensitivity with perceptual abilities (e.g., using similar tasks, but without illusions). Although the illusions used in the present study did differ in terms of the perceptual task (contrast-based, size-estimation, angle-perception), the possibility of our general factor being driven by inter-individual perceptual skills variability (or other cognitive skills) cannot be discarded. Moreover, using only the error rate models to extract individual-level scores might fail in capturing the whole range of behavioral dynamics. Future work should attempt at integrating the reaction times data (e.g., by jointly analyzing them using drift diffusion models), and assess the psychometric properties - such as stability (e.g., test-retest reliability) and validity - of similar illusion-based paradigms. Finally, while the personality measures used in this study highlight illusion sensitivity as an interesting measure rather than a mere perceptual artifact, further studies should test its relationship with more specific dispositional characteristics (e.g., autistic or schizotypal traits), cognitive styles and abilities, to help understand the potential underlying mechanisms of these associations.

## Data Availability

The datasets generated and/or analysed during the current study are available in the GitHub repository https://github.com/RealityBending/IllusionGameValidation.

## References

[1] Carbon C-C (2014). Understanding human perception by human-made illusions. Front. Hum. Neurosci..

[2] Day RH (1972). Visual spatial illusions: A General Explanation: A wide range of visual illusions, including geometrical distortions, can be explained by a single principle. Science.

[3] Eagleman DM (2001). Visual illusions and neurobiology. Nat. Rev. Neurosci..

[4] Gomez-Villa A, Martín A, Vazquez-Corral J, Bertalmío M, Malo J (2022). On the synthesis of visual illusions using deep generative models. J. Vis..

[5] Caporuscio C, Fink SB, Sterzer P, Martin JM (2022). When seeing is not believing: A mechanistic basis for predictive divergence. Conscious. Cognit..

[6] Lamme VAF (2020). Visual functions generating conscious seeing. Front. Psychol..

[7] Notredame C-E, Pins D, Deneve S, Jardri R (2014). What visual illusions teach us about schizophrenia. Front. Integr. Neurosci..

[8] Gori S, Molteni M, Facoetti A (2016). Visual illusions: An interesting tool to investigate developmental dyslexia and autism spectrum disorder. Front. Hum. Neurosci..

[9] Razeghi R, Arsham S, Movahedi A, Sammaknejad N (2022). The effect of visual illusion on performance and quiet eye in autistic children. Early Child Dev. Care.

[10] Teufel C (2015). Shift toward prior knowledge confers a perceptual advantage in early psychosis and psychosis-prone healthy individuals. Proc. Natl. Acad. Sci..

[11] Hamburger K (2016). Visual illusions based on processes: New classification system needed. Perception.

[12] Cretenoud AF, Francis G, Herzog MH (2020). When illusions merge. J. Vis..

[13] Halpern SD, Andrews TJ, Purves D (1999). Interindividual variation in human visual performance. J. Cogn. Neurosci..

[14] Thurstone, L. L. A factorial study of perception. (1944).

[15] Grzeczkowski L, Clarke AM, Francis G, Mast FW, Herzog MH (2017). About individual differences in vision. Vis. Res..

[16] Grzeczkowski L (2018). Is the perception of illusions abnormal in schizophrenia?. Psychiatry Res..

[17] Park S, Zikopoulos B, Yazdanbakhsh A (2022). Visual illusion susceptibility in autism: A neural model. Eur. J. Neurosci..

[18] Zhang Y (2017). Personality traits and perception of Müller-Lyer illusion in male Chinese military soldiers and university students. Transl. Neurosci..

[19] Konrath S, Bushman BJ, Grove T (2009). Seeing my world in a million little pieces: Narcissism, self-construal, and cognitive-perceptual style. J. Pers..

[20] Cretenoud AF (2019). Factors underlying visual illusions are illusion-specific but not feature-specific. J. Vis..

[21] Teufel C, Dakin SC, Fletcher PC (2018). Prior object-knowledge sharpens properties of early visual feature-detectors. Sci. Rep..

[22] Friston K (2010). The free-energy principle: A unified brain theory?. Nat. Rev. Neurosci..

[23] Adams RA, Stephan KE, Brown HR, Frith CD, Friston KJ (2013). The computational anatomy of psychosis. Front. Psych..

[24] Koethe D (2009). Binocular depth inversion as a paradigm of reduced visual information processing in prodromal state, antipsychotic-naive and treated schizophrenia. Eur. Arch. Psychiatry Clin. Neurosci..

[25] Bressan P, Kramer P (2021). Most findings obtained with untimed visual illusions are confounded. Psychol. Sci..

[26] Lányi, O., Keri, S., Pálffy, Z. & Polner, B. Can you believe your eyes? Positive schizotypy is associated with increased susceptibility to the müller-lyer illusion. (2022). 10.1016/j.schres.2023.12.023 38215568

[27] Coren S, Girgus JS, Erlichman H, Hakstian AR (1976). An empirical taxonomy of visual illusions. Percept. psychophys..

[28] Mylniec A, Bednarek H (2016). Field dependence, efficiency of information processing in working memory and susceptibility to orientation illusions among architects. Pol. Psychol. Bull..

[29] Cretenoud AF, Grzeczkowski L, Bertamini M, Herzog MH (2020). Individual differences in the müller-lyer and ponzo illusions are stable across different contexts. J. Vis..

[30] Cretenoud AF (2021). How do visual skills relate to action video game performance?. J. Vis..

[31] Skottun BC, Skoyles JR (2014). Subjective criteria and illusions in visual testing: Some methodological limitations. Psychol. Res..

[32] Cretenoud AF, Grzeczkowski L, Kunchulia M, Herzog MH (2021). Individual differences in the perception of visual illusions are stable across eyes, time, and measurement methods. J. Vis..

[33] Makowski D, Lau ZJ, Pham T, Paul Boyce W, Annabel Chen SH (2021). A parametric framework to generate visual illusions using python. Perception.

[34] Sibley C (2011). The mini-IPIP6: Validation and extension of a short measure of the big-six factors of personality in new zealand. N. Z. J. Psychol..

[35] Hopwood CJ, Thomas KM, Markon KE, Wright AGC, Krueger RF (2012). DSM-5 personality traits and DSM–IV personality disorders. J. Abnorm. Psychol..

[36] De Leeuw JR (2015). jsPsych: A JavaScript library for creating behavioral experiments in a web browser. Behav. Res. Methods.

[37] Peer E, Rothschild D, Gordon A, Evernden Z, Damer E (2022). Data quality of platforms and panels for online behavioral research. Behav. Res. Methods.

[38] Balota DA, Yap MJ (2011). Moving beyond the mean in studies of mental chronometry: The power of response time distributional analyses. Curr. Dir. Psychol. Sci..

[39] Matzke D, Wagenmakers E-J (2009). Psychological interpretation of the ex-gaussian and shifted wald parameters: A diffusion model analysis. Psychon. Bull. Rev..

[40] R Core Team. *R: A language and environment for statistical computing*. (R Foundation for Statistical Computing, 2022).

[41] Bürkner P-C (2017). brms: An R package for Bayesian multilevel models using Stan. J. Stat. Softw..

[42] Wickham H (2019). Welcome to the tidyverse. J. Open Sour. Softw..

[43] Makowski D, Ben-Shachar M, Lüdecke D (2019). bayestestR: Describing effects and their uncertainty, existence and significance within the Bayesian framework. JOSS.

[44] Makowski D, Ben-Shachar M, Patil I, Lüdecke D (2020). Methods and algorithms for correlation analysis in R. JOSS.

[45] Lüdecke D, Ben-Shachar M, Patil I, Waggoner P, Makowski D (2021). performance: An R package for assessment, comparison and testing of statistical models. JOSS.

[46] Lüdecke D, Waggoner P, Makowski D (2019). Insight: A unified interface to access information from model objects in R. JOSS.

[47] Makowski D, Ben-Shachar MS, Chen SA, Lüdecke D (2019). Indices of effect existence and significance in the bayesian framework. Front. Psychol..

[48] Lüdecke D, Ben-Shachar M, Patil I, Makowski D (2020). Extracting, computing and exploring the parameters of statistical models using R. JOSS.

[49] Bürkner P-C, Charpentier E (2020). Modelling monotonic effects of ordinal predictors in bayesian regression models. Br. J. Math. Stat. Psychol..

[50] Yang E (2012). Visual context processing in schizophrenia: Clinical. Psychol. Sci..

[51] Fox, A. Adolescent self-development and psychopathology: Anorexia nervosa and psychosis. (2006).

[52] Ohmann K, Burgmer P (2016). Nothing compares to me: How narcissism shapes comparative thinking. Personality Individ. Differ..

[53] Pessoa VF, Monge-Fuentes V, Simon CY, Suganuma E, Tavares MCH (2008). The müller-lyer illusion as a tool for schizophrenia screening. Rev. Neurosci..

